# Supplementary material for: Retinal and Choroidal Thinning—A Predictor of Coronary Artery Occlusion?
Source: Diagnostics (Basel). 2022 Aug 20;12(8):2016. doi: 10.3390/diagnostics12082016 (PMC9407460; doi:10.3390/diagnostics12082016)
Supplement: Supplementary file 1 [file diagnostics-12-02016-s001.zip › diagnostics-1860157-supplementary.pdf]

SUPPLEMENTS

Table S1. Comparison of retinal and choroidal thickness parameters between groups.

	**Healthy**<br>**Median (range)**	**MI group**<br>**Median (range)**	**3VD group**<br>**Median (range)**
**Retinal thickness**			
Central	249.5 (205.0-313.0)	249.5 (194.0-298.0)	246.0 (212.0-363.0)
Inner circle	310.9 (278.8-350.0)[1]	305.1 (272.5-341.8)[1]	308.0 (270.5-325.0)
Outer circle	272.3 (239.0-306.0)[2, 3, 4]	263.8 (236.5-301.5)[3, 4]	263.0 (229.0-280.8)[2, 3]
Statistical significance: 1 – *p=0.044,* 2 – *p=0.010;* 3 – *p=0.013;* 4 – *p=0.021.*			
**RNFL layer**			
Central	6.0 (1.0-20.0)	7.0 (1.0-22.0)	7.0 (2.0-19.0)
Inner circle	27.0 (21.5-44.3)	27.6 (21.5-62.3)	28.5 (22.8-49.3)
Outer circle	41.3 (32.5-58.8)	39.6 (30.3-78.0)	41.0 (32.0-62.3)
**GCL++ layer**			
Central	57.0 (34.0-103.0)	58.0 (20.0-81.0)	57.0 (41.0-78.0)
Inner circle	117.1 (97.8-146.3)	115.6 (92.5-144.3)	115.6 (97.0-131.0)
Outer circle	106.3 (91.5-134.5)	104.3 (85.3-142.8)	103.9 (81.3-113.5)
**GCL+ layer**			
Central	50.0 (32.0-85.0)	50.0 (27.0-68.0)	49.0 (32.0-63.0)
Inner circle	89.8 (72.3-112.5)[5, 6, 7]	85.9 (70.5-107.5)[5, 7]	87.4 (63.3-97.3)[5, 6]
Outer circle	65.5 (52.5-83.3)[8, 9]	62.8 (50.3-77.0)[8, 10]	60.4 (44.3-68.3)[8, 9, 10]
Statistical significance: 5 – *p=0.011;* 6 – *p=0.018;* 7 – *p=0.010,* 8 – *p=0.002;* 9 – *p<0.001;* 10 – *p=0.012.*			
**Choroidal thickness**			
Central	252.5 (43.0-427.0)[11]	252.5 (51.0-453.0)	207.0 (90.0-362.0)[11]
Inner circle	253.5 (123.5-407.8)[12, 14]	252.6 (65.5-443.3)[13, 14]	195.8 (96.8-327.0)[12, 13, 14]
Outer circle	224.0 (107.0-364.0)[15, 17]	231.5 (73.5-376.3)[16]	181.3 (92.8-291.8)[15, 16, 17]

Statistical significance: 11 – *p=0.035;* 12 – *p=0.005;* 13 – *p=0.011;* 14 – *p=0.016;* 15 – *p=0.002,* 16 – *p=0.003;* 17 – *p=0.004.*

The number next to the value represents groups being compared.

Table S2. Comparison of foveal avascular zone (FAZ) parameters between groups. Expressed as median (min.-max.). No statistically significant differences observed.

	**Healthy**	**MI group**	**3VD group**
3x3			
Superficial FAZ area, μm^2	282.74 (0-611.28)	284.55 (48.45-886.29)	308.85 (93.87-580.43)
Superficial FAZ vertical diameter, µm	600.0 (0.0-872.0)	579.5 (220.0-1153.0)	619.0 (380.0-926.0)
Superficial FAZ horizontal diameter, µm	565.0 (0.0-935.0)	610.5 (233.0-1012.0)	619.0 (249.0-899.0)
Deep FAZ area, μm^2	227.46 (41.57-716.22)	248.16 (44.12-700.84)	309.38 (86.31-760.61)
Deep FAZ vertical diameter, µm	577.0 (219.0-927.0)	538.5 (214.0-1028.0)	592.0 (316.0-966.0)
Deep FAZ horizontal diameter, µm	593.0 (225.0-983.0)	581.5 (245.0-1016.0)	663.0 (330.0-1004.0)
6x6			
Superficial FAZ area, μm^2	243.98 (54.49-608.91)	267.71 (46.58-721.76)	303.05 (74.18-511.52)
Superficial FAZ vertical diameter, µm	570.0 (235.0-881.0)	592.0 (116.0-947.0)	609.0 (329.0-817.0)
Superficial FAZ horizontal diameter, µm	545.0 (235.0-908.0)	571.5 (160.0-962.0)	614.0 (249.0-926.0)
Deep FAZ area, μm^2	225.70 (55.55-788.91)	250.49 (21.45-795.59)	300.94 (107.23-768.87)
Deep FAZ vertical diameter, µm	551.0 (245.0-944.0)	552.0 (160.0-981.0)	582.0 (338.0-939.0)
Deep FAZ horizontal diameter, µm	607.0 (246.0-1077.0)	600.0 (116.0-1237.0)	619.0 (365.0-1017.0)

Table S3. Comparison of vascular density in superficial and deep capillary plexus between groups. Expressed as median (min.-max.).

	Healthy	MI group	3VD group
**6x6**			
**Superficial capillary plexus**			
Central	21.30 (10.87-28.09)*	20.50 (7.60-30.71)	18.43 (11.75-25.63)
Nasal	45.72 (40.41-51.03)*	45.20 (35.73-50.84)	45.21 (40.05-48.78)
Temporal	45.78 (41.44-53.76)	45.86 (40.78-53.50)	44.95 (40.38-49.37)
Superior	48.30 (37.08-52.20)	47.97 (42.72-53.11)	46.76 (41.78-54.00)
Inferior	46.94 (41.16-52.98)	47.18 (40.14-57.40)	46.97 (35.41-49.83)
**Deep capillary plexus**			
Central	19.70 (9.31-27.15)	18.76 (6.82-30.35)	17.94 (10.70-25.20)
Nasal	49.02 (43.47-52.93)	48.44 (35.24-54.72)	49.04 (44.25-51.79)
Temporal	48.84 (43.37-59.80)	48.77 (42.20-54.59)	47.80 (41.66-52.68)
Superior	51.27 (37.66-55.95)	51.40 (45.44-57.28)	50.69 (43.06-55.95)
Inferior	50.46 (43.51-54.33)	49.90 (42.27-60.08)	50.44 (37.76-53.58)
**3x3**			
**Superficial capillary plexus**			
Central	21.12 (10.81-36.19)	20.25 (5.75-27.57)	18.82 (10.47-26.99)
Nasal	46.64 (40.33-51.28)	46.57 (41.14-54.31)	46.31 (42.38-49.69)
Temporal	46.91 (40.69-50.83)	46.91 (42.97-52.78)	46.08 (42.23-50.63)
Superior	49.54 (41.01-55.30)	48.95 (41.41-54.08)	48.60 (44.35-52.02)
Inferior	49.10 (37.21-55.60)	48.26 (35.80-59.53)	47.68 (36.98-52.88)
**Deep capillary plexus**			
Central	20.08 (10.56-35.15)	19.29 (4.3-29.39)	18.22 (10.00-27.14)
Nasal	50.38 (44.36-52.24)	50.03 (43.92-57.97)	49.49 (43.77-52.76)
Temporal	50.05 (42.18-54.26)	49.89 (45.18-56.19)	49.30 (45.26-53.99)
Superior	52.87 (43.01-62.77)	52.42 (43.35-58.77)	52.71 (44.44-55.68)
Inferior	51.91 (40.44-60,47)	51.91 (38.62-62.12)	51.63 (38.83-55.38)

*Statistically significant difference comparing healthy and 3VD group.

No other statistically significant differences observed.
